# A brief overview of 20 years of neuroscience in PLoS Computational Biology

**DOI:** 10.1371/journal.pcbi.1014468

**Published:** 2026-07-06

**Authors:** Hugues Berry, Lyle J. Graham, Kim T. Blackwell

**Affiliations:** 1 AIstroSight, Inria, Hospices Civils de Lyon, Université Claude Bernard Lyon 1, Villeurbanne, France; 2 Centre Giovanni Borelli—CNRS UMR, Université Paris Cité, Paris, France; 3 Roy J. Carver Department of Biomedical Engineering, University of Iowa, Iowa City, United States of America; 4 Iowa Neuroscience Institute, University of Iowa, Iowa City, United States of America; Johns Hopkins University, UNITED STATES OF AMERICA

## Introduction

Neuroscience has been a major domain of *PLoS Computational Biology* since its birth, spanning a wide range of topics, including systems biology applied to the brain, detailed biophysical modeling, neural network models, network analysis, image analysis methodologies, models for behavior and cognition, or AI-based analysis and comparisons with real data. Clearly, the themes tackled cover the realm of computational neuroscience in a broad sense.

On the occasion of the celebration of *PLoS Computational Biology*’s 20th anniversary, we reflect on what has been the positioning of neuroscience and its evolution in the journal. How many articles each year have dealt with neuroscience? What are the main classes of questions raised in these neuroscience articles?

Our analysis shows that the number of neuroscience articles has increased over the past 20 years at the same rate as the total number of articles in the journal, representing a stable 23% of the total articles. Maybe unsurprisingly, the institution of the last authors of these articles have been mainly located in the USA, with a significant number from the UK and Germany. Furthermore, we notice a clear evolution of the questions addressed across this period, with a move from cellular-level models, such as biophysical and reaction-diffusion models, to systems-wide and whole-brain scales. Moreover, the advent of new domains appears clearly in recent years, including the application of computational models to brain pathologies, the emergence of large databases of models and data, AI, and studies focused around ethics or policy. We conclude with a brief perspective.

## Quantitative analysis

**Fig 1:**
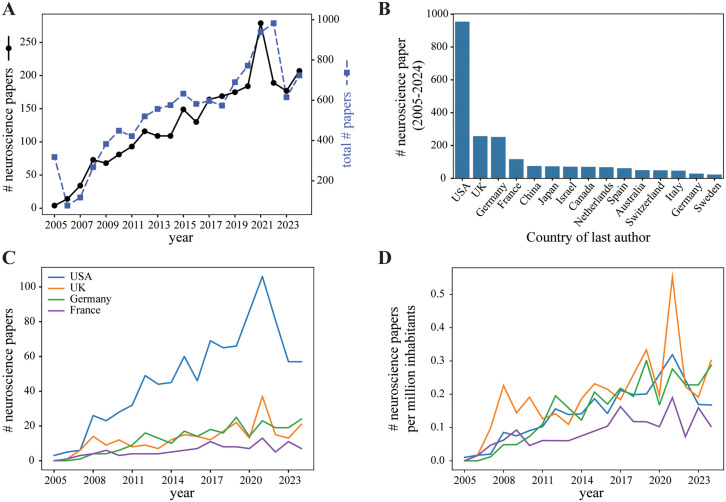
Descriptive analysis of the neuroscience articles published in PLoS Computational Biology. The number of neuroscience articles published each year is compared to the total number of articles in the journal **(A)**. The distribution of neuroscience papers by country (2005–2024) restricted to the 15 countries with largest counts is shown in **(B)**. Note that here “country“ designates the country declared in the last affiliation of the last author. Time courses show the raw number of papers **(C)** or the number of papers by million inhabitants **(D)** for the first 4 countries of **(B)**.

The number of papers dedicated to neuroscience has been steadily increasing since the launch of the journal (Fig 1A, *black circles+full line*). This increase was particularly strong at the beginning, until 2015, with the count subsequently tending to stabilize, at around 180 published articles per year. Strikingly, the total number of articles published in the journal (whatever the topic), followed almost the exact same trend (Fig 1A, *blue squares+dashed line*). As a result, the fraction of neuroscience articles has been rather stable over the last 20 years, with 1 neuroscience article for every 4–5 articles (average of 22.6 ± 6.6% neuroscience articles every year).

The last authors of these neuroscience articles were predominantly based in the USA, and, more rarely, in the UK and Germany (Fig 1B and 1C). However, a different picture appears when scaling these numbers with the population of each country (Fig 1D). This reveals that the UK, USA, and Germany have similar publication rates when normalized by population, in contrast to France, for example, which has fewer publications relative to population.

## Content and topic analysis

The focus of neuroscience publications in *PLoS Computational Biology* has evolved similar to the field as a whole. Namely, with the advent of omics, and advances in imaging approaches and *in vivo* recording techniques, there has been a shift from cellular and circuit level studies, to the analysis and modeling of omics data, as well as leveraging *in vivo* recording to investigate more complex behaviors.

In the journal’s first decade (2005–2014), the neuroscience articles of *PLoS Computational Biology* mainly concerned the exploration of cellular and circuit-level studies. Many of the cellular studies investigated mechanisms underlying various types of synaptic plasticity, including spike-timing-dependent plasticity. A number of articles also focused on biophysical and detailed models of single neurons, for example, trying to explain firing patterns from ion channel properties. Circuit-level studies evaluated, for example, how different types of plasticity mechanisms could explain working memory, attention, and decision making, as well as the functional circuits underlying visual processing, sensorimotor integration, or auditory processing. Other subjects that gained prominence in the first decade were related to neuroimaging techniques (fMRI, EEG, connectivity). These publications typically used graph theory, dynamical systems, and other abstract network models to explain fMRI data.

Neuroscience focii in the second decade (2015–2024) moved quite significantly. Whereas multisensory integration continued to attract much interest, new topics emerged, in particular reflecting advances in experimental neuroscience. For example, as deep brain stimulation included treatments of more clinical conditions, and with the improvement of multi-channel recordings, computational studies began investigating how EEG data could be used for brain-computer interfaces. Likewise, with more experiments focused on resting–state fMRI, more *PLoS Computational Biology* studies were concerned with resting-state dynamics. The increasing availability of compute power has yielded studies leveraging large-scale brain models to predict the effect of pharmacotherapy on EEG dynamics, or predicted structural connectivity from fMRI. Similarly, an increase in computational omics-based studies has mirrored the expansion of omics databases. The overall growing availability of large databases has also included other types of data, including electrophysiological and anatomical, in all cases being supported by increasingly sophisticated ecosystems for analysis and modeling. In parallel, the sorts of behaviors being modeled has swelled to include complex cognitive tasks going beyond the levels of integration implicated in decision making or perception. Domains associated with these questions have included social cognition (empathy, theory of mind), emotion processing (stress, rewards, mood), ethics (specifically neuroethics), and finally consciousness. In addition, this last decade witnessed a vigorous move towards pathology and diseases (mostly neurodegenerative diseases or neuroinflammation). Finally, with the expansion of open access policies, we see the appearance of publications related to data sharing and reproducibility.

## AI in neuroscience articles

In recent years, the advent of AI and deep/machine learning has been a major event in *PLoS Computational Biology* neuroscience articles, as is the case for many other venues. Our general editorial policy has been to publish articles where the AI methodology is used to uncover new understandings of the studied biological system, brain or nervous system. As such, we have excluded articles whose goal is primarily to explain the operation of an artificial AI system (though exceptions have been made when potential applications to a biological system are clear and outstanding). Indeed, presenting a convincing argument that the way a particular artificial neural network solves a task is similar to the way a real nervous system solves it is generally quite difficult, demanding more than a comparison of a few statistics on how the task is solved by the two systems, artificial and biological.

Some of the applications of AI and machine learning in the published neuroscience articles are not necessarily limited to neuroscience. These include the analysis of single-cell and spatial omics (gene regulatory network inference, cell–cell interactions, multimodal integration) or structural biology studies (protein-ligand binding, 3D structures, incl. chromatin). Some applications are, however, more specific to neuroscience. This includes population analyses, where the studies focus on the analysis and modeling of neural population codes, including spike trains, energy-related questions, or on the analysis of behavioral data. A large group of AI neuroscience studies also tackles topics related to scientific computing, automation and model pipelines in neuroscience. This encompasses the proposal of methodologies for, e.g., automated reconstruction of neural activity recordings, parameter calibration for detailed neuron models, or pipelines for the processing of experimental data (cleaning, segmentation, reconstruction).

## Conclusion and perspectives

Though the ratio of neuroscience-related articles has been quite stable throughout the journal’s lifespan, their domains of interest have strongly moved.

i *From biophysics to AI*: the focus has moved from traditional biophysical or cognitive models (e.g., spike-timing, ion channel-based) to deep learning and AI-based methodologies.
ii *From cellular scales to systems levels*: recent works tend to prefer large-scale brain networks (resting-state, whole-brain models) over studies related to isolated regions or isolated circuits.
iii *Moving towards the clinics*: the concern has strongly shifted toward the study of diseases, with a special focus on neurodegenerative and affective disorders
iv *The emergence of ethics and open science*: a new emphasis appeared related to ethical, policy or reproducibility themes, and the availability of large amounts of open source data and open access codes.

Given the rate at which practically every technological field has been evolving—in particular that of computer-based fields, predicting where the neuroscience domain will go in the next 20 years of *PLoS Computational Biology* can be considered as rather speculative. Of course, one can easily gauge that applications to pathologies will be increasingly important in the future, if only because of the utility to society as a whole. By the same token it seems likely that applications related to prevention, or more generally on how to keep a healthy status (health studies), will become even more prominent. With the advent of more powerful data analysis architectures and algorithms, neurotechnologies are also likely to be more prominent in the journal (e.g., brain-computer interfaces). Likewise, one may dream that within the next 20 years, it may become increasingly possible to integrate in a single model all the levels of spatiotemporal scales that define neurobiology: molecular, cellular, regional, whole-brain, and behavioral. This might be instrumental to help develop integrated theories for higher brain functions (personality, mind, consciousness…) and to test them. Whether these few ideas will indeed become new hot topics sometime in the next 20 years is a very risky bet that we are averse to predict. The only thing we can do is to invite our readers to meet again in 2045 to see what exciting neuroscience developments emerged meanwhile in *PLoS Computational Biology*!

## Methods

The journal has no computer records of its papers published before 2017, when the current Editorial Management information system came in use. Therefore, we fetched the 2005–2024 PLoS Computational Biology articles from Pubmed using the E-utilities API. To filter out neuroscience articles, we searched for mesh keywords containing neur*, or brain, or glia*, or astrocyte*. This yielded 2,524 abstracts for the period January 2005–December 2024. The total number of articles published in PLoS Computational Biology in the period was retrieved from SciSpace ([href:https://scispace.com/journals/plos-computational-biology-278ha2gu]https://scispace.com/journals/plos-computational-biology-278ha2gu). A synthetic sematic analysis of the content and main topics of the abstracts of the neuroscience articles was carried out using ChatGPT ([href:https://chatgpt.com/]https://chatgpt.com/, model version GPT-5.1) followed by a careful examination of the results based on human examination of a randomly selected subset of the abstracts. The normalization publication counts by population was based on data from ChatGPT ([href:https://chatgpt.com/]https://chatgpt.com/, model version GPT-5.1) and was spot checked with several population databases.

